# Salt in Sickness

**Published:** 1874-04

**Authors:** 


### SALT IN SICKNESS.

A man apparently in the advanced stages of consumption became very fond of salt, so much so that he carried it in his vest pocket, which was replenished every morning ; he took a pinch at a time and allowed it to dissolve in his mouth ; he used a tablespoonful or more of it every day. He recovered, with good health afterwards.

In any form of sickness, if there is a craving for salt, it may be safely considered as an indication that nature demands that element, and that the ailment is, in part at least, owing to the want of it. In some forms of sores and wounds which are slow to heal, or which give out a bad odor, a very free use of salt alone, or on the food, will often cause a remarkable change for the better in in a few days, there is no repugnance in its use.

Salt prevents meats from putrifying. Common salt consists of one equivalent of sodium and one of chlorine, an element which in all its combinations is purifying and antagonistic of decay. A teaspoonful of salt every fifteen or twenty minutes is often efficacious in arresting bleeding from the lungs; several such doses in a day rapidly diminishes enlarged spleen. It has been used in Hungary many years as a good substitute for quinine in cases of intermittent fever. A pound of salt dissolved in four gallons of water gives the strength of sea water, and is as good as a sea bath.

